# Cell Type Variability in the Incorporation of Lipids in the Dengue Virus Virion

**DOI:** 10.3390/v14112566

**Published:** 2022-11-19

**Authors:** Atitaya Hitakarun, Maia Kavanagh Williamson, Nathamon Yimpring, Wannapa Sornjai, Nitwara Wikan, Christopher J. Arthur, Julien Pompon, Andrew D. Davidson, Duncan R. Smith

**Affiliations:** 1 Institute of Molecular Biosciences, Mahidol University, Salaya, Bangkok 73170, Thailand; 2 School of Cellular and Molecular Medicine, University of Bristol, Bristol BS81TD, UK; 3 Department of Pharmacology, Faculty of Medicine, Chiang Mai University, Chiang Mai 50200, Thailand; 4 School of Chemistry, Faculty of Science, University of Bristol, Bristol BS81TD, UK; 5 Maladies Infectieuses et Vecteurs: Ecologie, Génétique, Evolution et Contrôle (MIVEGEC), Institut de Recherche Pour le Développement (IRD), University Montpellier, CNRS, 911, Avenue Agropolis, 34394 Montpellier, France

**Keywords:** dengue virus, glycerolipids, sphingolipids, glycerophospholipids, LLC-MK2 cells, C6/36 cells, virion, lipid

## Abstract

A lipid bilayer produced from the host membrane makes up around 20% of the weight of the dengue virus (DENV) virion and is crucial for virus entry. Despite its significance, the virion’s lipid composition is still poorly understood. In tandem with lipid profiles of the cells utilised to generate the virions, this work determined a partial lipid profile of DENV virions derived from two cell lines (C6/36 and LLC-MK_2_). The results showed distinctive profiles between the two cell types. In the mammalian LLC-MK_2_ cells, 30.8% (73/237 identified lipid species; 31 upregulated, 42 downregulated) of lipid species were altered in response to infection, whilst in insect C6/36 cells only 12.0% (25/208; 19 upregulated, 6 downregulated) of lipid species showed alterations in response to infection. For virions from LLC-MK_2_ cells, 14 lipids were detected specifically in virions with a further seven lipids being enriched (over mock controls). For virions from C6/36 cells, 43 lipids were detected that were not seen in mock preparations, with a further 16 being specifically enriched (over mock control). These results provide the first lipid description of DENV virions produced in mammalian and mosquito cells, as well as the lipid changes in the corresponding infected cells.

## 1. Introduction

Viruses of the genus *Flavivirus* (family *Flaviviridae*) are a major public health concern worldwide with nearly the entire human population at risk of infection [1]. The genus contains a number of well-known mosquito-transmitted human pathogenic viruses distributed in tropical and subtropical countries including yellow fever virus (YFV), dengue virus (DENV), Japanese encephalitis virus (JEV), Zika virus (ZIKV) and West Nile virus (WNV) [2], as well as a number of lesser known viruses with the potential to emerge such as Wesselsbron virus and St. Louis encephalitis virus [3]. Highly effective human vaccines exist for only two of the approximately 30 human pathogenic mosquito-transmitted viruses, namely YFV [4] and JEV [5], while a partially effective vaccine against DENV is only recommended for patients between 9- and 45-years-old who have already been infected by one of the four DENV serotypes [6]. There is currently no specific drug to treat the diseases caused by any of the viruses in this genus and vector control is only partially effective in preventing epidemics [7].

Flaviviruses have a positive sense single-stranded RNA genome of approximately 11 kb that encodes a single open reading frame that is translated into one polypeptide before being cleaved by the host and virally encoded proteases into three structural proteins (Capsid (C), pre-membrane (prM) and envelope (E)) and seven non-structural (NS) proteins (NS1, NS2A, NS2B, NS3, NS4A, NS4B and NS5) [8]. The non-structural proteins form the replication complexes to enable viral genome replication and blunt host cell innate immune responses [9].

The flavivirus virion is composed of a nucleocapsid core surrounded by a lipid bilayer in which 180 copies of the (post cleavage) membrane and E proteins are embedded [10,11]. The lipid bilayer is derived from host cell membranes in which the E protein becomes embedded during viral replication [11]. Little is known about the composition of the lipid bilayer, which is surprising considering its function in the virus replication cycle and its role in mediating interactions between cellular receptors and virus entry [12,13]. However, it has been estimated that, for DENV, there are approximately 8000 lipid molecules [14] that comprise some 20% of the weight of the virion. Early studies also estimated the lipid content of Sindbis virus from the related genus *Alphavirus* (originally classified together with the genus *Flavivirus* as belonging to the family *Togaviridae*) to be as high as 30% dry weight [15]. Interestingly, it has been shown for Semliki Forest virus (SFV) (another mosquito-transmitted alphavirus) that the lipid content of the mature virion differed when the virus was grown in mosquito or mammalian cells [16].

Lipids are broadly defined as organic compounds that are insoluble in water, but are soluble in organic solvents, and a number of different classification systems have been developed [17]. The International Lipid Classification and Nomenclature Committee developed a comprehensive system (LIPID MAPS) that divides lipids into eight categories (fatty acyls (FA), glycolipids (GL), glycerophospholipids (GP), sphingolipids (SP), saccharolipids (SL), polyketides (PK), sterol lipids (ST) and prenol lipids (PR)), with each category being further divided into (at least) classes and subclasses [18,19].

Only one study, using WNV as a model, has applied modern analytical methodologies such as liquid chromatography-mass spectrometry to describe the lipid content of flaviviruses [20]. The authors reported that certain SP were found to be enriched in the lipid envelope of the virus, concomitant with an increase of the same SP in infected mammalian cells [20]. However, different flaviviruses have different lipid requirements, as has been shown for the differential impact of SP on WNV and DENV multiplication [21], and as such the previous study may not be reflective of the DENV lipid composition.

In this study, we examined the lipid content of DENV virions, the most common flavivirus, by analysing the lipid profile for three categories of lipids (SP, GP and GL) and using the appropriate mock-infected purified controls. To test whether virion composition differs when grown in insect or mammalian cells, we analysed viruses produced from the insect *Aedes albopictus* C6/36 cell line [22] and the mammalian rhesus monkey kidney LLC-MK_2_ cell line [23]. To determine if the virion composition reflects infection-induced changes in the cells, we also performed a similar analysis on the cells from which the viruses had been grown.

## 2. Materials and Methods

### 2.1. Cells

C6/36 cells (ATCC CRL-1660) were cultured at 28 °C in minimum essential medium (MEM, GIBCO, Invitrogen. Grand Island, NY, USA) supplemented with 10% foetal bovine serum (FBS) and 100 units/mL of penicillin/streptomycin (GIBCO, Invitrogen. Grand Island, NY, USA). LLC-MK_2_ (ATCC CCL-7 [23]) cells were cultured at 37 °C with 5% CO_2_ in Dulbecco’s modified eagle’s medium (DMEM, GIBCO, Invitrogen. Grand Island, NY, USA) supplemented with 5% FBS and 100 units penicillin/streptomycin per mL.

### 2.2. Large Scale Virus Preparation

DENV serotype 2 (strain 16681; [24]) was separately propagated at a large scale in both C6/36 and LLC-MK_2_ cells, representative of mosquito and mammalian cells, respectively. Both sets of viruses were independently grown and purified in triplicate with parallel mock infections in 5 T175 flasks. After infection at a multiplicity of infection of 1 or mock infection, the cells were cultured at 28 °C and 37 °C for C6/36 and LLC-MK_2_, respectively. On day 3 (LLC-MK_2_) or day 6 (C6/36) post infection, the supernatant of the infected cells was harvested, and viruses were precipitated with 10% (*w*/*v*) PEG 8000 and 1.5 M NaCl at 4 °C overnight. After centrifugation, the viral pellet was resuspended in TNE buffer (10 mM Tris-HCl, pH 7.5, 140 mM NaCl, 1 mM EDTA) containing 10% (*w*/*v*) sucrose, layered onto a discontinuous sucrose step gradient of 30 and 60% sucrose (*w*/*v*) (providing a 10/30/60 step gradient at the start of centrifugation) and centrifuged at 82,705× *g* for 3.5 h. After centrifugation, the fraction immediately above the 60% sucrose cushion was collected, diluted with TNE and viruses were pelleted by ultracentrifugation at 82,705× *g* for 1.5 h in an SW28 rotor (Beckman, Fullerton, CA, USA) before re-suspension in TNE buffer containing 1% bovine serum albumin. Virus recovery was confirmed by plaque assay on LLC-MK_2_ cells as described previously [25]. The cells used to propagate the viruses were also collected by scraping, pelleted by centrifugation, and stored at −80 °C before use. All work using live viruses was undertaken in a BSL2 laboratory after approval by the Institutional (Mahidol University, Bangkok, Thailand) Biosafety Committee.

### 2.3. Lipid Extraction

Lipids were extracted from whole cell pellets (~1 × 10^6^ cells) resuspended in 50 μL TNMg buffer (20 mM Tris-HCl [pH 8], 100 mM NaCl, 25 mM MgCl_2_) and from 10 µL of purified virions using a modified Bligh and Dyer [26] protocol (CHCl_3_:MeOH:H_2_O = 2:2:1 (*v*/*v*/*v*)). Briefly, CHCl_3_ was added to all samples, which were then incubated on ice for 1 h. MeOH and H_2_O were added sequentially, and samples were incubated at 15 °C with agitation. A further volume of CHCl_3_ was added and samples incubated for a further 15 min before centrifugation at 13,500× *g* for 10 min at 15 °C. The resulting lower organic fraction containing extracted lipids was collected. Additional volumes of CHCl_3_ were then added to the remaining aqueous fraction, which were incubated for 30 min with agitation, centrifuged and a second extraction performed. Organic extracts were combined and dried under a N_2_ stream. Samples were stored at −80 °C until analysis.

### 2.4. Lipid Analysis

Dried lipid samples were reconstituted and external standards added to all samples: CE(18:0-d6), C15:0-d29 FA, C17:0-d33 FA, C20:0-d39 FA, TG(45:0-d29), TG(48:0-d31), TG(54:0-d35) (all from CDN isotopes), C16-d31 ceramide, PA(C16:0-d31/C18:1) Na^+^ salt, PC(C16:0-d31/C18:1), PE(C16:0-d31/C18:1), PG(C16:0-d31/C18:1) Na^+^ salt, PI(C16:0-d31/C18:1) NH4^+^ salt, PS(C16:0-d62) Na^+^ salt and SM(C16:0-d31) (all Avanti Polar Lipids). Lipid analysis was performed at the Core Metabolomics and Lipidomics Laboratory (CMaLL) at the University of Cambridge. Chromatographic separation was achieved using a Waters Acquity UPLC CSH C18 (50 mm × 2.1 mm, 1.7 μm) LC-column with a Shimadzu UPLC system (Shimadzu UK Limited, Wolverton, Milton Keynes, UK). The column was maintained at 55 °C with a flow rate of 0.5 mL/min. A binary mobile phase system was used with mobile phase A acetonitrile:water mix (6:4, respectively, with 10 mM ammonium formate), and mobile phase B isopropanol:acetonitrile mix (9:1, respectively, with 10 mM ammonium formate). The gradient profile was as follows: at 0 min 40% mobile phase B, at 0.4 min 43% mobile phase B, at 0.45 min 50% mobile phase B, at 2.4 min 54% mobile phase B, at 2.45 min 70% mobile phase B, at 7 min 99% mobile phase B, at 8 min 99% mobile phase B, at 8.3 min 40% mobile phase B, at 10 min 40% mobile phase B. Mass spectrometry detection was performed on a Thermo Exactive orbitrap mass spectrometer (Thermo Scientific. Hemel Hempstead, UK) operating in positive ion and negative ion continuous switching mode. Ions were generated using a heated electrospray source; the sheath gas was set to 40 (arbitrary units), the aux gas set to 15 (arbitrary units) and the capillary temperature set to 300 °C. Mass spectra were recorded in full scan mode from *m*/*z* 150–1200 Da. Lipid species were identified based on both their retention time and accurate mass and quantified relative to the external standards.

### 2.5. Normalisation and Statistical Analysis

Perseus (version 1.6.6.0, Max Planck Institute of Biochemistry, Planegg, Germany [27]) was used to analyse the data. For analysis of the cellular lipids the data were log2 transformed and normalised by subtracting the sample mean (global mean scaling) to account for heteroskedasticity. Statistical significance and fold changes were calculated using a Welch’s two-tailed *t*-test and corrected for multiple testing using the Benjamini–Hochberg false discovery rate method, with sample presence required in two samples from each experimental condition. Comparative analysis of the virion lipids was performed by subtracting the amounts of each lipid determined in the mock samples from the corresponding virion sample. Each lipid was then expressed as a percentage of the total lipid for that sample and significant differences in lipid composition determined using a Welch’s two-tailed *t*-test with correction for multiple testing using the Benjamini–Hochberg false discovery rate method.

### 2.6. cDNA Synthesis by RT-PCR

Viral RNA was extracted from the supernatant of DENV 2-infected C6/36 cells using a QIAamp Viral RNA Mini Kit (QIAGEN, Hilden, Germany) following the manufacturer’s protocol. Isolated DENV 2 RNA was used as a template for cDNA synthesis by reverse transcription. Briefly, viral RNA was incubated with 2.5 µM random hexamers (Thermo Fisher Scientific, Waltham, MA, USA) at 95 °C for 2 min and cDNA was subsequently synthesized in a reaction mix containing 1X reaction buffer, 1 mM dNTPs, 1 unit of RiboLock RNase Inhibitor and 10 units of RevertAid Reverse Transcriptase (Thermo Fisher Scientific, Waltham, MA, USA). The reaction was incubated at 25 °C for 5 min, 42 °C for 90 min and 70 °C for 10 min using a Veriti Thermo Cycler (Applied Biosystems, Foster City, CA, USA).

### 2.7. RNA Standard Preparation

A portion of the DENV 2 NS5 gene was amplified in a PCR reaction containing cDNA template, 1X Phusion HF buffer, 200 μM dNTPs, 200 nM DENV 2 NS5-T7 forward primer (5′-TAATACGACTCACTATAGGGCTCCCTGAGTGGAGTGGAAG-3′), 200 nM of DENV 2-NS5 reverse primer (5′-ACACGCACCACCT-TGTTTTG-3′) and Phusion high fidelity DNA polymerases (Thermo Fisher Scientific, Waltham, MA, USA). The reaction was carried out under the following conditions: 98 °C for 30 sec followed by 30 cycles of denaturation at 98 °C for 10 sec, annealing at 65 °C for 30 sec, extension at 72 °C for 10 sec and a final elongation at 72 °C for 5 min. PCR products were run on a 2% agarose gel. The agarose gel portion containing the NS5 PCR product was cut out and purified using FavorPrep GEL/PCR purification kit (FAVORGEN Biotech Corporation, Ping-Tung, Taiwan) according to the manufacturer’s protocol. The purified DENV 2 NS5 DNA was used as a template for in vitro RNA transcription using an Invitrogen MEGAscript T7 Transcription Kit (Thermo Fisher Scientific, Waltham, MA, USA) following the manufacturer’s protocol. The transcribed DENV 2 NS5 RNA was purified using E.Z.N.A. total RNA kit I (Omega Bio-tek, Norcross, GA, USA) and contaminating DNA was removed using a RapidOut DNA Removal Kit (Thermo Fisher Scientific, Waltham, MA, USA). RNA concentration was measured using a Nanodrop2000 and the RNA copy number was calculated by the Science Primer web calculator (http://www.scienceprimer.com/copy-number-calculator-for-realtime-pcr (accessed on 20 July 2022)). Synthesized DENV 2 NS5 RNA was kept at −80 °C as a standard for one-step qRT-PCR.

### 2.8. One Step qRT-PCR

Viral RNA was extracted from mock or DENV 2-purified samples using a QIAamp Viral RNA Mini Kit (Qiagen, Hilden, Germany) following the manufacturer’s protocol. DENV 2 gene copy number of purified RNA samples and 10-fold serial diluted DENV 2 NS5 RNA standards was quantitated using iTaq universal SYBR Green one-step kit (Bio-Rad, Hercules, CA, USA) and a Mastercycler Realplex (Eppendorf, Hauppauge, NY, USA). The amplification reaction contained RNA template, 1X iTaq universal SYBR green reaction mix, 300 nM DENV 2 NS5 forward primer (5′-CTCCCTGAGTGGAGTGGAAG-3′), DENV 2-NS5 reverse primer (5′-ACACGCACCACCTTGTTTTG-3′) and iScript reverse transcriptase. The reaction was carried out under the following conditions: reverse transcription at 50 °C for 10 min, followed with an initial denaturation at 95 °C for 1 min and 40 cycles of denaturation at 95 °C for 10 sec and annealing coupled with extension at 60 °C for 45 sec. DENV 2 gene copy number was calculated based on the standard curve generated from the Ct cycle of the RNA standard.

### 2.9. Western Blot Analysis

A total of 5 µL of sucrose gradient purified DENV 2 virion and virion mock control prepared from both C6/36 and LLC-MK_2_ cells and 5 µL of protein prepared from the retained cell pellets were boiled and subsequently separated by electrophoresis through 10% SDS polyacrylamide gels. Proteins were transferred to nitrocellulose membranes using a Trans-Blot electrophoretic transfer cell (Bio-Rad Laboratories Inc., Hercules, CA, USA). The membranes were blocked with 5% skim milk (*w*/*v*) in TBS-T containing 0.05% Tween-20 with gentle shaking at room temperature for 15 min. After that, the membranes were washed once with TBS-T for 5 min. Proteins were detected by incubating the membranes with a 1:500 dilution of a pan specific mouse monoclonal anti-flavivirus E protein antibody (HB112 (4G2)), a 1:1000 dilution of a rabbit polyclonal anti-Alix primary antibody (ab88388; Abcam, Cambridge, UK), or a 1:10,000 dilution of a rabbit polyclonal anti- Hsp90 α/β (sc-7947; Santa Cruz Biotechnology Inc., Dallas, TX, USA) in 5% skim milk in 1X TBS-T as primary antibodies with gentle shaking at 4 °C for overnight. The membranes were incubated with a 1:8000 dilution of a horseradish peroxidase conjugated goat anti-rabbit IgG antibody (Pierce), or a 1:500 dilution of a horseradish peroxidase (HRP) conjugated goat anti-mouse IgG with gentle shaking at room temperature for 1 h. Signals were developed using the Immobilon Forte Western HRP Substrate and immediately detected using a ChemiDoc XRS + system running controlled by Image Lab software (Bio-Rad Laboratories Inc., Hercules, CA, USA).

## 3. Results

### 3.1. Purification of Viral Particles

We first confirmed virion purification by titering the virus in the supernatants from infected C6/36 and LLC-MK2 cells, and the corresponding purified virions. Titers of purified virions from C6/36 cells ranged from 1.45 × 10^9^ to 1.8 × 10^10^ pfu/µL, while purified virions from LLC-MK_2_ cells ranged from 1.075 × 10^7^ to 1.75 × 10^8^ pfu/µL (Table S1). Although titers of supernatants used for purification were homogenous between insect and mammalian cells, purification efficiency was lower for LLC-MK_2_-produced virions (Table S1). We next established the DENV 2 genome copy number in the purified samples (both virion and mock) by one step qRT-PCR. None of the mock samples had any amplification product, while genome copy number in the purified virion samples ranged from 2.25 × 10^9^ to 7.91 × 10^10^ for the C6/36 generated samples, and from 7.50 × 10^7^ to 3.52 × 10^9^ for the LLC-MK_2_ generated samples (Table S2). The results from the genome copy number analysis were consistent with the results observed by plaque assay. We next undertook a western blot analysis of all samples, which confirmed the presence of E protein in all virion prep samples, and no signal was seen in the mock preparations (Figure S1).

**Figure 1:**
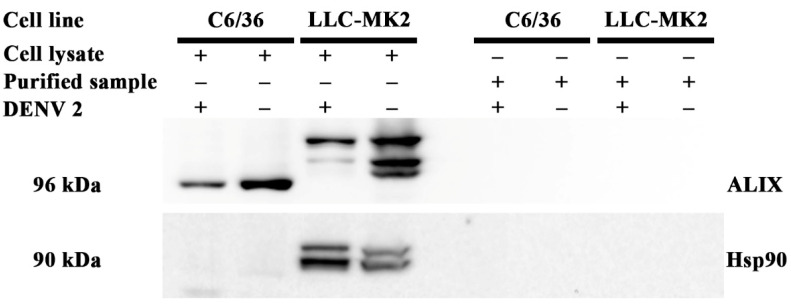
Detection of exosome protein markers in purified virus samples. The supernatants from mock and DENV infected C6/36 and LLC-MK_2_ cells were separated through a 10/30/60% (*w*/*v*) discontinuous sucrose gradient. The exome markers ALIX and Hsp90 were detected in the purified preparations and in lysates of infected cells used for the preparations by western blot analysis.

We next evaluated whether the purified samples from the mock infections and the DENV infections contained contaminating exosomes, which largely comprised lipids, through the detection of ALIX and Hsp90, which are believed to be cell type independent markers of exosomes [28]. The proteins in lysates from both C6/36 and LLC-MK_2_ cells were used as the positive controls for the detection of ALIX and Hsp90. Figure 1 shows the detection of ALIX in both the C6/36 and LLC-MK_2_ cell lysates, but not in the purified virion preparation or the mock control preparation. A marked size difference was observed in the ALIX signal between C6/36 cells and LLC-MK_2_ cells. According to the UniProt database [29], human ALIX is 868 amino acids long, while insect ALIX (in this case *Drosophila melanogaster*) is 836 amino acids long, which is consistent with the results shown. In addition, multiple bands were seen for the LLC-MK_2_ ALIX protein. While the identity of the multiple bands is not known, it is likely they represent yet uncharacterized isoforms. Hsp90 was only detected in the lysate of LLC-MK_2_ (mammalian) cells (Figure 1) possibly because the predominant stress chaperone protein of insect cells is Hsp70 [30]; however, the antibody failing to detect insect Hsp90 is also a possibility. Again, no signal was observed in the virion preparations or the mock infection preparations. Altogether, our data indicate that the preparations were highly concentrated in virions and devoid of exosomes.

### 3.2. DENV Infection Alters the Cellular Lipid Profile

**Figure 2:**
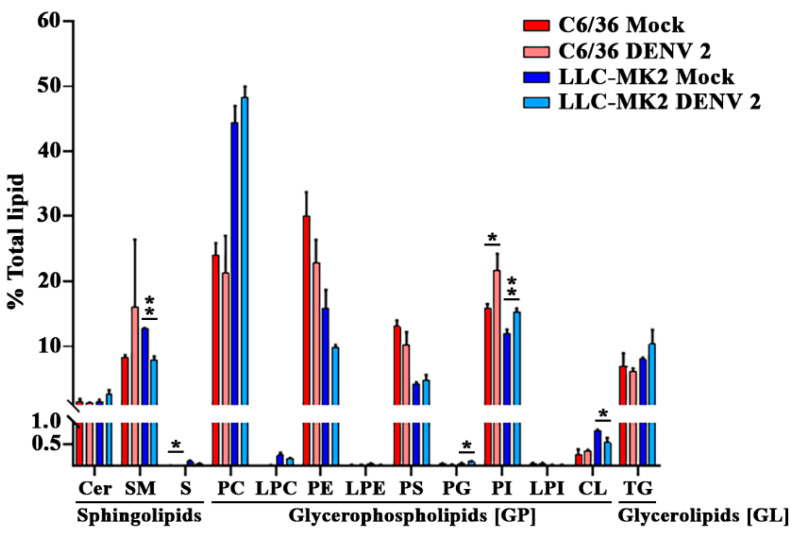
Different cell types display distinct lipid profiles. The lipid composition of whole cell lysates from mock and DENV 2 infected C6/36 and LLC-MK_2_ cells are shown grouped by LIPIDSMAPS class. Statistical significance determined by Welch’s 2-tailed *t*-test, * *p* ≤ 0.05, ** *p* ≤ 0.01. Data are from three biological replicates. Bars show average + S.D. Cer (Ceramide), SM (Sphingomyelin), S (Sulfoglycosphingolipids), PC (Phosphatidylcholine), LPC (Lyso-PC), PE (Phosphatidylethanolamine), LPE (Lyso-PE), PS (Phosphatidylserine), PG (Phosphatidylglycerol), PI (Phosphatidylinositol), LPI (Lyso-PI), CL (Cardiolipin), TG (Triglyceride).

In infected and mock cells, a total of 275 lipids from the SP, GP and GL categories were detected and quantified in relative amounts as compared to the standards (Table S2). Analysis of the mock infected samples revealed a distinct lipid profile for each cell type, differing significantly in the relative composition of lipids from each class (Figure 2). Most notably, the predominant lipid class in C6/36 mock infected cells was phosphatidylethanolamine (PE) compared with phosphatidylcholine (PC) for LLC-MK_2_ cells. A total of 200 lipids were detected in mock-infected cells from both cell types, with 15 additional lipids present in infected C6/36 cells and 53 in infected LLC-MK_2_ cells (Table 1 and Table S3). The lipid profile of each cell type changed in response to DENV infection. For C6/36 cells, significant alterations were observed in sulfoglycosphingolipids (S) and phosphatidylinositol (PI) classes, whilst for LLC-MK_2_ cells significant alterations were observed in SM, PI, PG and CL lipid classes (Figure 2).

**Figure 3:**
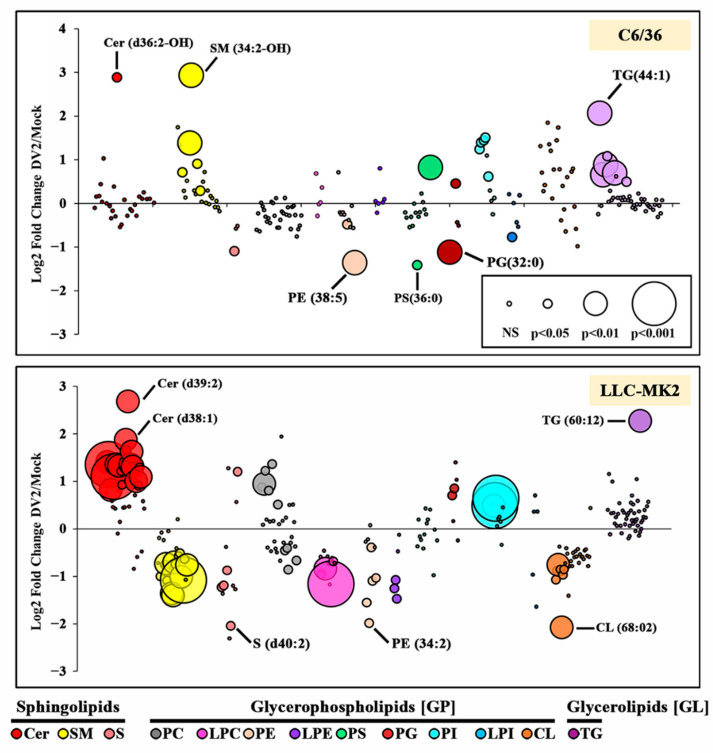
Log2 fold change of lipid species in DENV 2 infected compared with mock control cells. Bubbles represent individual species analysed in biological triplicate and normalised by global mean scaling. Bubble size represents significance of the difference as determined by two-tailed Welch’s *t*-test. Lipid classes are shown as separate colours and arranged in increasing summed chain length from left to right. Total lipids shown C6/36 cells = 208 and LLC-MK_2_ cells = 237. Significant changes in C6/36 cells = 25 and LLC-MK_2_ cells = 73. Cer (Ceramide), SM (Sphingomyelin), S (Sulfoglycosphingolipids), PC (Phosphatidylcholine), LPC (Lyso-PC), PE (Phosphatidylethanolamine), LPE (Lyso-PE), PS (Phosphatidylserine), PG (Phosphatidylglycerol), PI (Phosphatidylinositol), LPI (Lyso-PI), CL (Cardiolipin), TG (Triglyceride).

To investigate these changes further, we compared the relative abundance of the individual lipid species between DENV 2 and mock-infected cells (Figure 3). Within C6/36 cells, 208 lipids were identified in both mock- and DENV 2-infected cells (in at least two of three replicates, Table S4), of which 25 changed significantly (12%) (19 upregulated and 6 downregulated, Table 1) The largest fold upregulated lipids were SM (34:2-OH), Cer (d36:2-OH) and TG (44:1), whilst the largest fold downregulated lipids were PS (36:0), PE (38:5) and PG (32:0) (Figure 3, Table S4). Within the SM and TG classes, significant alterations were only observed in lipids with a low summed carbon composition. Specifically, detected SM lipids ranged from 30–44 carbons but significant changes were only detected in lipids with ≤38 carbons. Similarly, the detected TG lipids ranged from 36 to 66 carbons, but only lipids ≤50 carbons showed significant changes. There were no significant changes in the CL, LPC, LPE or PC lipid classes in response to DENV infection.

Within LLC-MK_2_ cells, 237 lipids were detected in both mock- and DENV-infected cells (in at least two of the three replicates, Table S5), of which 73 (30.08%) were significantly altered in response to infection (31 upregulated and 42 downregulated, Table 2). The largest fold upregulated lipids were Cer (d39:2), TG (60:12) and Cer (d38:1), whilst the largest fold downregulated lipids were CL (68:02), S (d40:2) and PE (34:2) (Figure 3, Table S5). It is notable that more than 60% of Cer and SM lipids were significantly altered in response to DENV 2 infection. With the exception of PS and LPI, significant alterations were observed in all lipid classes.

In the PE, PI, PG and SM classes, a small number of lipid species were significantly altered in both C6/36 and LLC-MK2 cells, including lipid species that were altered between DENV and mock infections in the same direction (e.g., both upregulated or both downregulated; Table 2) and in the opposite direction (one upregulated, one downregulated; Table 2).

In addition to changes in lipid abundance, we also detected lipid species that were only present in mock- or DENV-infected cells (Table 3). The lipid species PI (35:0) was absent from both C6/36 and LLC-MK_2_ cells but present in their DENV 2-infected counterparts. Conversely, S (d33:1) and (d36:1) were present in mock samples from both cell types, but not detected after infection with DENV 2. It is of note that the lipids shown in this table often represent some of the least abundant lipids in cellular samples. As a result, their presence or absence in samples could represent only a minor change in their abundance, pushing them above or below the detection limit. By contrast, the lipid PE (36:3) was within the 10% of most abundant lipids in LLC-MK_2_ mock-infected cells and yet was undetectable in DENV 2-infected cells.

### 3.3. Lipid Profiles of DENV Virions

Lipid extraction and analysis of virion samples revealed that lipids were present in both the mock and virion preparations, as previously reported for influenza virus [31]. While the source of these lipids in the mock preparations is unknown, they likely represent carryover from lipids in the cell growth medium not eliminated by the purification process. The presence of lipids in the mock underscores the importance of this control. The mock preparations from both cell types were composed of >98% of TG lipids with near 100% identity between cell types. High proportions of the virion preparations were also composed of TG lipids (77% and 97.6% of C6/36 and LLC-MK_2_ virion preparations, respectively). The lipid composition of buffers, FBS and a no cell control extraction from plasticware also showed a high concentration of TG lipids. Finally, TG lipids were excluded from this lipidomic analysis because no significant differences in TG lipids were observed between mock and virions preps (data not shown).

For C6/36 cells, 43 lipid species were only detected in virion preparations and not mock preparations, with a further 16 lipid species significantly enriched in the virion preparations. For LLC-MK_2_ cells, 14 lipids were only detected in virion preparations, with seven species significantly enriched in the virion preparation (Table 4 and Table S6). Analysis of the lipids either enriched or only found in virions (from C6/36 and LLC-MK_2_ cells) compared to the mock preparations identified a larger variety of PC (17 species for C6/36, and seven species for LLC-MK_2_) as compared to other lipids. Other PL species from the PG, PI and PS classes were also detected in relatively large numbers, while lower numbers of Cer and CL species were detected in virions produced in mammalian and insect cells. Notably, no lysophospholipids were detected, although they are involved in the formation of replication complexes [32]. Insect-produced virions also contained PE and SM species. Interestingly, seven species (1 CL, 2 PC, 1 PG, 2 PI and 1 PS) were commonly detected in virions produced from both cells.

**Figure 4:**
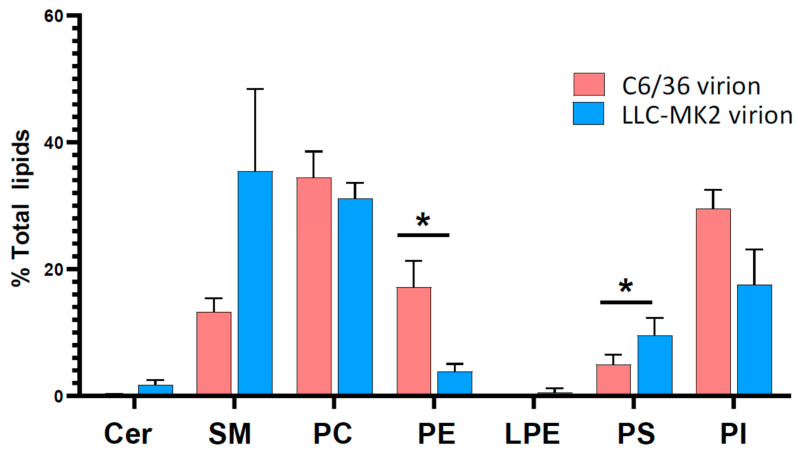
Profile of lipid classes from virion preparations from C6/36 and LLC-MK_2_ cells. The amounts of background lipids detected in mock preparations were subtracted from the amounts detected in virion preparations from DENV 2-infected C6/36 and LLC-MK_2_ cells. Each lipid was represented as the % of total lipid and then grouped by LIPIDSMAPS class as shown. Classes in which the summed lipid % contribution <0.2 of the total lipid are not shown to simplify the data set. Bars show average + S.D. Statistical significance determined by Welch’s 2-tailed *t*-test, * *p* ≤ 0.05. Data from three biological triplicates. Cer (Ceramide), SM (Sphingomyelin), PC (Phosphatidylcholine), PE (Phosphatidylethanolamine), LPE (Lyso-PE), PS (Phosphatidylserine), PI (Phosphatidylinositol).

Analysis of the composition of lipid classes in the virion preparations from C6/36 and LLC-MK_2_ cells, after subtracting the background values from mock preparations, revealed distinct lipid profiles (Figure 4, Table S7). There were significant differences in the lipid classes PE and PS as a percentage of total lipid in virions from DENV 2-infected C6/36 and LLC-MK_2_ cells. PS contributed to a greater percentage and PE a lesser percentage of the total lipids in virions from LLC-MK2 cells compared to virions for C6/36 cells, respectively. For the lipid classes Cer, PE and PI, the proportion of each lipid class in the virion preparations correlated with the relative proportions of the lipid classes in the cells from which the virions were derived. PE lipids appeared to constitute a greater proportion of the total lipid in both C6/36 cells (DENV 2-infected and mock) and virions produced from them compared to LLC-MK_2_ cells. By contrast, there were marked differences in the lipid classes SM, PC and PS as a percentage of total lipids, between the virions and the cells from which they were derived (Figure 2 and Figure 4). Specifically, SM and PC lipids formed a greater percentage of the total lipids in virions derived from LLC-MK_2_ cells compared to the cellular lipid profile.

## 4. Discussion

The mature flavivirus virion is known to contain about 20% lipid by weight [12] that is derived from the host cell. While several studies have investigated the changes in cellular lipid content during flavivirus infection [33,34,35,36], only one study on WNV has analysed the lipid content of the virion [20]. Markedly, while that study also looked at cellular changes, they did not include a virion “mock” control, and thus there is some uncertainty in their interpretation. As noted in this study, the mock virion controls showed the presence of a significant amount of lipids.

In this study we infected two different cell lines, namely C6/36 cells that are derived from *Aedes albopictus* whole larvae [22], and an LLC-MK_2_ cell line derived from rhesus monkey kidneys [23]. These two cell lines represent both aspects of the flavivirus replication cycle (insect vector and mammalian host). We performed the analysis at later time points than most studies (3 days post-infection (dpi) for LLC-MK_2_ and 6 dpi for C6/36) to profile lipids of cells with productive infection and established virion secretion. The virus was propagated, purified and used for lipid extraction, as were host cell lipids from the infected cells. The lipid samples were analysed by LC-MS. Only three (SP, GP and GL) of the eight LIPID MAPS categories [18,19] were analysed, but two of these categories (GL, SL) are two of the three categories that contain the majority of lipids found in mammalian cell membranes [37]. The third major category, sterols, requires specific analytical techniques and, like fatty acyls, was not included in this study. Insect cell membranes have been reported to be profoundly different from mammalian cell membranes, with one study showing 51% dissimilar membrane lipids comparing between BHK 21 cells and *Aedes albopictus* cells [38], although this is not reflected in the category of lipids present.

The two cell lines had distinct lipid profiles according to analysis of the three lipid categories we selected (SP, GP and GL). In particular, nearly 50% of total lipids identified in LLC-MK_2_ cells were PC glycerophospholipids, which were present at only slightly more than 20% of total lipids in the insect cell line. This difference in lipid content between mammals and insects has previously been observed [39]. With the exception of Cer and TG, changes in cellular lipid composition were observed in response to infection for nearly every lipid class detected. There were 200 lipid species found to be common to both cell lines, but LLC-MK_2_ cells had a greater number of unique lipid species (53) than insect cells (13). How exactly DENV infection alters the host cell lipidome remains largely unknown, but we have previously shown that the expression of enzymes involved in lipogenesis, lipolysis and fatty acid oxidation is dysregulated [40,41], which would obviously have an effect on the cellular lipidome. Lipids are important at many stages of the viral replication cycle [13,41], and we have previously shown that inhibiting fatty acid synthase, a critical lipid regulatory enzyme, significantly reduces viral replication [25,40].

For the lipid categories examined (SP, GP and GL) mammalian LLC-MK2 cells exhibited more remodelling in response to infection than C6/36 cells. While 30% (73/237 identified lipid species; 31 upregulated, 42 downregulated) of lipid species in the three selected categories (SP, GP and GL) were altered in response to infection in LLC-MK_2_ cells, only 12% (25/205; 19 upregulated, 6 downregulated) of lipid species in insect C6/36 cells showed alterations in response to infection. This suggests that viral replication in insect cells requires less remodelling of lipids in the three analysed categories (SP, GP and GL) than occurs in mammalian cells to achieve effective viral replication. Interestingly, only five lipid species were seen as altered in the same way in both cell lines. Overall, there were very significant differences, with the most discordant results being observed for SL. While Cer sphingolipids showed a significant degree of upregulation in LLC-MK_2_ cells, these were largely unchanged in C6/36 cells, with one exception (d36:2-OH). Several SM sphingolipids were upregulated in C6/36 cells but predominantly downregulated in mammalian LLC-MK2 cells. Lipid remodelling is required for the formation of the replication complex in both mammalian and mosquito cells via endoplasmic reticulum invagination. [32,41]. The major structural lipids in eukaryotic membranes are glycerophospholipids: phosphatidylcholine (PC), phosphatidylethanolamine (PE), phosphatidylserine (PS), phosphatidylinositol (PI) and phosphatidic acid (PA), Sphingolipids (particularly Cer) and sterols (which were not analysed in this study (particularly cholesterol)) [42]. We discovered a significant regulation for multiple classes of structural lipids that form membranes and endomembranes in our study, and these regulations may reflect the lipid reconfiguration required to form replication complexes. While replication complex structures appear similar in both mammals and mosquitoes [43,44], surrounding membranes appear different, which could explain the difference in lipid reconfiguration observed between LLC-MK2 and C6/36 cells.

After excluding probable contaminants (e.g., TG), there was still a relatively large number of lipid classes in the three selected categories (SP, GP and GL) seen in both the preparations from the mock and the virion preparations. This suggests that lipids associated with copurifying proteins, non-exosome extracellular vesicles or cellular debris may serve to provide some background in the analysis. While Cer sphingolipids were seen to be broadly upregulated in LLC-MK_2_ cells, the same was not seen in virion preps, where Cer sphingolipids were present as a low percentage of total lipids for both virions prepared from LLC-MK_2_ cells, and from C6/36 cells. In insect Aag2 cells Cer has been shown to be required for DENV replication [33], but consistent with this study only very few species show changes in abundance in response to infection. Comparison of LLC-MK_2_ and C6/36 virion preparations with mock preparations, identified a number of specific Cer sphingolipids, indicating their inclusion in the virion membrane. Despite the large enrichment of Cer sphingolipids in LLC-MK_2_ cells as a consequence of infection, only one Cer species (d40:2) was found to be significantly increased (or uniquely present) in virions from LLC-MK_2_ cells. Furthermore, no SM sphingolipids were detected as significantly enriched or uniquely present in LLC-MK_2_ virions compared to mock preparations, despite the finding that this lipid class composed ~35 % of the total virion lipid. The SM lipids SM 34:1 and 42:1 were the major contributors to the SM lipid class in the virions from LLC-MK_2_ cells. These findings suggest that the lipid enrichment observed in virions only represents a subset of the global lipidome changes observed in host cells, and that cell lipid reconfiguration does not solely serve the purpose of making envelope lipids available. Nonetheless, a number of species that were found in the virion preps were correspondingly enriched in infected mammalian (species enriched in both cells and virions: Cer d40:2; PC 30:0, 31:0, 32:0, 33:0; PG 36:1, PI 35:2, 36:1) and mosquito (species enriched in both cells and virions: PG 36:2; PI 34:1, 35:2, 36:1, 36:3; PS 40:4; SM 33:1, 34:2, 34:2-OH, 36:2) cells. Infection-mediated increase in these cellular lipid species may facilitate virus assembly by increasing the amount of the envelope lipids.

The PC lipid class comprised the greatest percentage of total lipids in virions from C6/36 cells and the greatest percentage of total lipids in virions from LLC-MK_2_ virions, after SM lipids. For both virion preparations PC lipids accounted for the highest number of species found in the virions that were either significantly enriched or unique compared to the mock preparations. Although other classes of PL including PE, PG and PI represented lower proportions of the overall lipid content than PC, these had a good number of species detected in both preparations. Of the eight lipid species commonly found in mammalian and insect produced virions, seven were PL. As PL interacts with cellular receptors for virus entry [45], these common PL may indicate the minimum lipid signature for effective virus infection. Apart from PL, Cer and CL were detected in both preparations, and SM lipids were unique mosquito-virions only compared to the mock preparations. The roughly spherical shape of DENV is produced by an adequate composition and distribution of lipids in the envelope [14]. Based on the cryo-EM structure of DENV virions a computational model of the DENV envelope was built using about 8000 molecules of PC, PE, PS, SM and Cer [14]. Our study indicates that CL and PG are also present in the DENV envelope and provides a detailed description of the lipid species present in virions produced from both insect and mammalian cells.

It is worth noting that 59 lipid species were found to be either uniquely present or significantly enriched in C6/36 virion preparations compared to mock, whereas only 21 lipid species were found to be enriched or unique in LLC-MK_2_ virions compared to mock. This would support slightly different membrane recruitment mechanisms in infected insect and mammalian cells. Our findings with Cer and SM sphingolipids contradict previous findings with WNV virions and recombinant subviral particles (RSPs) [20]. However, because the previous study did not include a virion “mock” control, its findings must be interpreted with caution. Another important difference between the Martin-Acebes study [20] and this study was in the propagation of viruses. In contrast to the previous study, where virions (and RSPs) were collected after the cells had been incubated for 48 h in serum-free medium, in the present study, virions were collected along with the cells that had been grown in normal growth media at either 3 or 6 days post-infection for LLC-MK_2_ and C6/36, respectively. Cell growth in serum free medium can impact cell viability and protein expression [46] as well as alter cellular oxidative stress [47], which could possibly affect the cellular lipidome. Lastly, the Martin-Acebes study [20] utilized HeLa cells, and as shown in this study the cell line utilized impacts the lipid profile of the mature virion. Given differences in methodology, virus and cell line the differences between the two studies must be interpreted with great caution.

Overall, our findings support cell type-specific lipidome remodelling as a result of infection. However, it should be borne in mind that the results for both cells and virions was based on three independent biological replicates, and as such some significant changes may have been missed due to sample variability and low statistical power. There is a cell origin specificity to the virion “lipidome” as well (as seen previously with the Alphavirus SFV [16]), but it does not reflect the global cellular lipid changes that are occurring. This theory contends that cellular regional changes occur in specific membranes within the cell, and that these changes are reflected in the virion lipid content. Furthermore, during virus assembly, there could be a selective process for lipid selection. Future research could look into how, or if, the different lipid incorporation into virions from different cell origins modulates the physiochemical properties of the virion. This study also suggests that targeting a small number of cellular lipids may be an effective antiviral strategy.

## Figures and Tables

**Table 1:** Lipid species identified in mock infected cells. The number of species from LIPIDMAPS classes detected commonly in mock-infected C6/36 and LLC-MK_2_ cells, as well as the number of lipid species from each class detected in only one cell type are shown. * in at least 2 of the 3 replicates.

		Cell Type Detected *		
Lipid Class	Full Name	C6/36 and LLC-MK_2_	C6/36 Only	LLC-MK_2_ Only
Cer	Ceramide	23	4	8
SM	Sphingomyelin	23	2	4
S	Sulfoglycosphingolipids	6	-	9
PC	Phosphatidylcholine	31	-	-
LPC	Lyso-PC	5	1	9
PE	Phosphatidylethanolamine	11	1	-
LPE	Lyso-PE	5	1	1
PS	Phosphatidylserine	13	2	3
PG	Phosphatidylglycerol	3	2	3
PI	Phosphatidylinositol	10	-	-
LPI	Lyso-PI	5	2	1
CL	Cardiolipin	23	-	9
TG	Triglyceride	42	-	6
Total		200	15	53

**Table 2:** Lipids significantly up- and downregulated during DENV 2 infection. Table shows lipid species that were significantly up-/downregulated in C6/36 and LL-MCK_2_ cells in response to DENV 2 infection. Lipid species that were significantly up-/downregulated in both cell types either in the same or opposite directions are shown in bold and denoted with an * or underlined respectively.

	C6/36: DENV 2 Infected/Mock		LLC-MK_2_: DENV 2 Infected/Mock	
Lipid Class	Upregulated	Downregulated	Upregulated	Downregulated
Cer	(d36:2-OH)		(d32:1), (d33:1), (d34:0), (d35:1), (d36:1), (d36:2), (d37:0-OH), (d37:1), (d38:1), (d39:1), (d39:2), (d40:1), (d40:2), (d41:2), (d42:2), (d43:1), (d43:1-OH), (d43:2),	
SM	(33:1), **(34:2)**, (34:2-OH), **(36:2)**, (38:0)			(32:1), (34:0-OH), (34:1), **(34:2)**, (35:2), (36:1), **(36:2)**, (37:1), (38:1), (40:1), (40:2), (41:1), (42:1), (42:2), (44:2)
S		(d34-OH:1)	(d44-OH:2)	(d32:1), (d34:1), (d36-OH:2), (d40:2)
PC			(30:0), (31:0), (32:0), (33:0), (34:0), (35:0),	(36:3), (37:2), (37:3), (40:3)
LPC				(16:1), (18:1), (20:0), (20:4)
PE		(36:1) *, (38:5) *		(32:1), (34:2), (36:1) *, (36:2), (38:5) *
LPE				(16:0), (18:0), (18:1)
PS	(40:4)	(36:0)		
PG	(36:2)	**(32:0)**	**(32:0)**, (36:1)	
PI	(34:1), (34:2) *, (35:2) *, (36:1) *, (36:3)		(34:2) *, (35:2) *, (36:1) *	
LPI		(18:0)		
CL				(66:04), (68:02), (68:03), (67:03), (68:04), (66:02), (66:03)
TG	(44:1), (45:1), (46:1), (46:2), (48:2), (50:3)		(60:12)	
Total	19	6	31	41

**Table 3:** Unique lipid species. Unique lipid species detected (in at least 2 of 3 replicates) only in mock- or DENV 2-infected C6/36 and LLC-MK2 cells.

	C6/36		LLC-MK_2_	
Lipid Class	Mock	DENV 2	Mock	DENV 2
Cer		(d40:0)	(d37:2)	(d33:1-OH)
				(d34:0-OH)
				(d36:0)
		(30:1)	(30:0)	
SM		(33:0)	(38:0)	
		(42:0)		
	(d32:1)	(d34:1)	(d33:1)	(d38:0)
S	(d33:1)	(d42:1)	(d36:1)	
	(d36:1)	(d42:2)	(d41:1)	
	(22:6)		(14:0)	
LPC			(17:1)	
			(18:2)	
PE			(36:3)	
LPE			(18:4)	
			(34:3)	
PS			(38:1)	
			(38:5)	
PG	(38:2)			
PI		(35:0)		(35:0)
LPI	(17:0)		(20:5)	(22:6)
CL	(74:10)	(66:02)	(74:11)	
		(75:05)		
TG		(45:2)	(56:1)	(62:12)
Total	7	11	17	7

**Table 4:** Alterations in the lipid species composition of virions compared to mock preparations. Table shows lipid species that were only detected in the virion but not mock preparations and lipid species that were significantly enriched in the virion compared to mock preparations, which are denoted with a *. Lipid species that were common between virions produced in insect and mammalian cells are in bold.

**C6/36 Lipid Species**								
**Cer**	(d34:1) *	**CL**	(66:03)	**PC**	(30:0)	(34:3)	**PE**	(36:2)
	(d36:1)		(66:04)		**(31:0)**	(34:4) *		
	(d36:2)		**(68:03)**		(32:1) *	(35:1)		
	(d38:1)		(68:04)		(32:2) *	(36:3) *		
	(d39:2)		(72:08)		**(33:0)**	(37:1)		
	(d40:1)				(33:2)	(38:1)		
	(d42:2)				(34:0)	(38:5) *		
					(34:1) *	(38:6) *		
					(34.2) *			
	(32:0)	**PI**	(34:1) *	**PS**	(32:1)		**SM**	(32:1)
	(34:1) *		**(35:2)**		(33:1)			(33:1)
	**(36:2)**		(36:1) *		(34:1)			(34:2) *
**PG**	(36:3)		(36:2)		**(38:1)**			(34:2-OH)
	(36:4)		(36:3)*		(38:5)			(36:0)
	(38:1)		(38:4)*		(38:6)			(36:2) *
	(38:2)				(40:4)			(38:1)
								(39:1)
								(40:2)
**LLC-MK_2_ Lipid Species**								
**Cer**	(d40:2) *	**CL**	**(68:03)**	**PC**	(30:0) *	(32:2)	**S**	**(d42:2)**
			(72:07)		(30:1)	**(33:0)**		
					**(31:0)**			
					(32:0) *			
					(32:1) *			
**PG**	(36:1)	**PI**	(34:1) *	**PS**	(36:1) *			
	**(36:2)**		**(35:2)**		(36:2)			
			(36:1) *		**(38:1)**			
			(36:4)		(40:2)			

## Data Availability

The data presented in this study are openly available in Zenodo at doi 10.5281/zenodo.7092598.

## Supplementary Materials

The following supporting information can be downloaded at: https://www.mdpi.com/article/10.3390/v14112566/s1, Figure S1: Western blot analysis of independent triplicates of purified virus and corresponding mock preparations [48]; Table S1: titer of infected supernatants and purified virions produced from C6/36 and LLC-MK2 cells and full length western blot; Table S2. DENV 2 gene copy number in purified samples in 3 replicates; Table S3: total cell analysis; Table S4: mock-infected cells; Table S5: C636_cells_DV2vMo; Table S6: LLCcells-DV2vMO; Table S7: total virion analysis.
