# Supplementary material for: Cell Type Variability in the Incorporation of Lipids in the Dengue Virus Virion
Source: Viruses. 2022 Nov 19;14(11):2566. doi: 10.3390/v14112566 (PMC9698084; doi:10.3390/v14112566)
Supplement: Supplementary file 1 [file viruses-14-02566-s001.zip › viruses-1952853-supplementary.pdf]

Figure S1, Tables S1 and S2 and uncropped western blots.

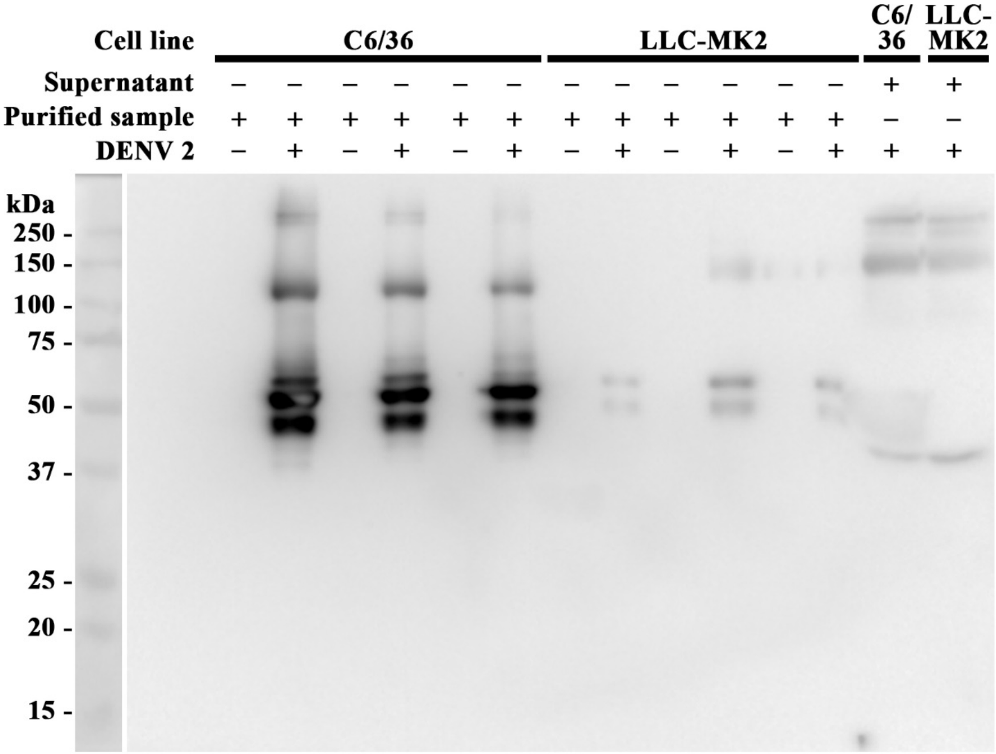

Supplemental Figure S1.

Western blot analysis of independent triplicates of purified virus and corresponding mock preparations. Samples were electrophoresed through 12% SDS PAGE gels after which proteins were transferred to solid matrix and probed with monoclonal antibody HB112 (mAB 4G2). Multiple bands of similar sizes have been observed by others using this antibody [1]. The whole, uncropped blot is shown together with the protein marker lane.

1. Zanluca, C.; Mazzarotto, G. A.; Bordignon, J.; Duarte Dos Santos, C. N., Development, characterization and application of monoclonal antibodies against Brazilian Dengue virus isolates. *PLoS One* **2014,** 9, (11), e110620.

Table S1. Titer of infected supernatants and purified virions produced from C6/36 and LLC-MK_2 cells.

Pfu, particle forming unit.

No.	Name	Supernatant (Before purification)		Purified sample		Concentration fold
		Volume (ml)	Titer (Pfu/μl)	Volume (μl)	Titer (Pfu/μl)	
1	D2L, C6/36-1	150	1.5x10^6	50	9.2x10^9	6,166
2	D2L, C6/36-2	150	2.7x10^8	50	1.8x10^10	66
3	D2L, C6/36-3	150	7.5x10^7	50	1.4x10^9	19
4	D2L, LLC-MK_2-1	150	2.3x10^7	50	1.0x10^7	0.46
5	D2L, LLC-MK_2-2	150	2.6x10^7	50	1.7x10^7	0.64
6	D2L, LLC-MK_2-3	150	3.5x10^7	50	1.7x10^8	5

Table S2. DENV 2 gene copy number in purified samples in 3 replicates

Samples	copies/μL	Sample Volume (μL)	Copies/sample
C6/36_Mock L.2	0	54	0
C6/36_Mock L.3	0	4	0
C6/36_Mock L.4	0	36	0
C6/36_DENV2 L.2	4.34×10^{7}	52	2.25×10^{9}
C6/36_DENV2 L.3	1.58×10^{9}	50	7.91×10^{10}
C6/36_DENV2 L.4	5.44×10^{8}	18	9.79×10^{9}
LLC_Mock L.1	0	28	0
LLC_Mock L.2	0	14	0
LLC_Mock L.4	0	4	0
LLC_DENV2 L.1	1.67×10^{7}	38	6.33×10^{8}
LLC_DENV2 L.2	1.26×10^{8}	28	3.52×10^{9}
LLC_DENV2 L.4	9.38×10^{6}	8	7.50×10^{7}

Uncropped western blots

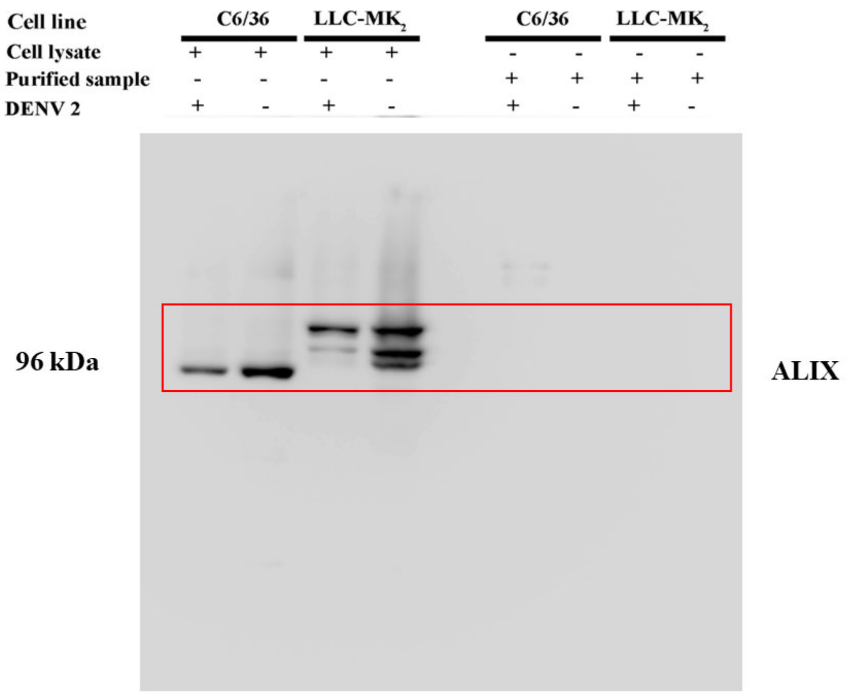

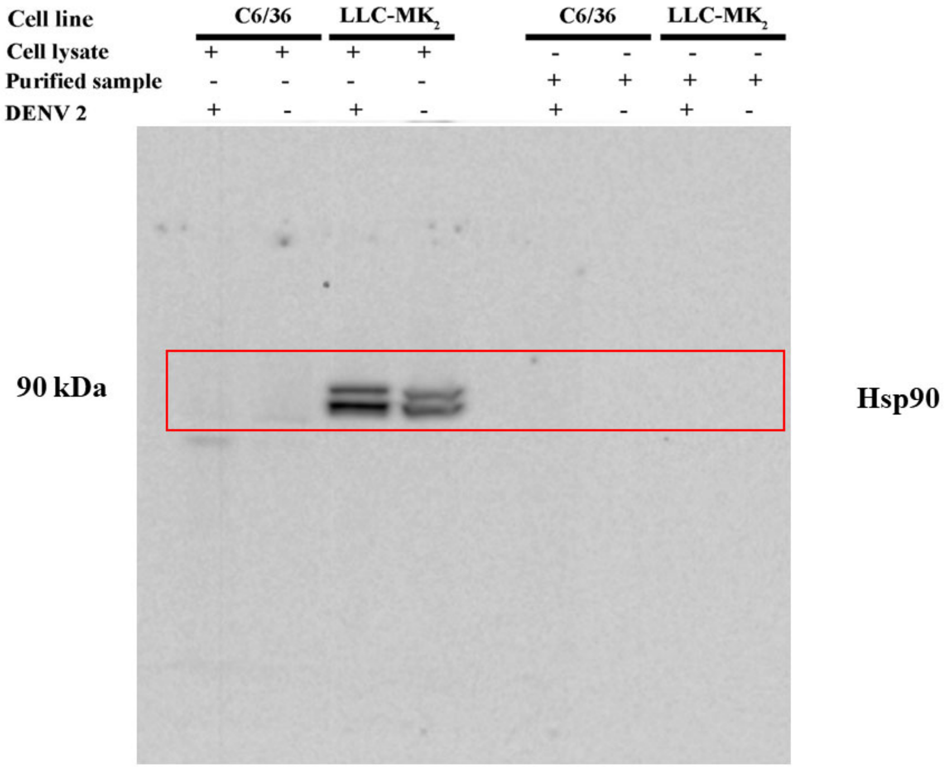

Cell line
C6/36
LLC-MK2
C6/36
LLC-MK2
Cell lysate
Purified sample
DENV 2
90 kDa
Hsp90
